# Genetic Relatedness and Novel Sequence Types of Non-O157 Shiga Toxin-Producing *Escherichia coli* Strains Isolated in Argentina

**DOI:** 10.3389/fcimb.2016.00093

**Published:** 2016-08-30

**Authors:** Jimena S. Cadona, Ana V. Bustamante, Juliana González, A. Mariel Sanso

**Affiliations:** Laboratorio de Inmunoquímica y Biotecnología, Facultad de Ciencias Veterinarias, Centro de Investigación Veterinaria de Tandil, Consejo Nacional de Investigaciones Científicas y Técnicas, Comisión de Investigaciones Científicas, Universidad Nacional del Centro de la Provincia de Buenos AiresTandil, Argentina

**Keywords:** non-O157 Shiga toxin-producing *Escherichia coli*, MLST, Argentinean clones, food-borne pathogen, zoonotic risk

## Abstract

Shiga toxin-producing *Escherichia coli* (STEC) is a foodborne pathogen responsible for severe disease in humans such as hemolytic uremic syndrome (HUS) and cattle, the principal reservoir. Identification of the clones/lineages is important as several characteristics, among them propensity to cause disease varies with STEC phylogenetic origin. At present, we do not know what STEC clones, especially of non-O157:H7, are circulating in Argentina. To fill this knowledge gap we assessed the genetic diversity of STEC strains isolated in Argentina from various sources, mostly cattle and food, using multilocus sequence typing (MLST). Our objectives were to determine the phylogenetic relationships among strains and to compare them with strains from different geographic origins, especially with those from clinical human cases, in order to evaluate their potential health risk. A total of 59 STEC isolates from 41 serotypes were characterized by MLST. Analysis using EcMLST database identified 38 sequence types (ST), 17 (45%) of which were new STs detected in 18 serotypes. Fifteen out of 38 STs identified were grouped into 11 clonal groups (CGs) and, 23 not grouped in any of the defined CGs. Different STs were found in the same serotype. Results highlighted a high degree of phylogenetic heterogeneity among Argentinean strains and they showed that several cattle and food isolates belonged to the same STs that are commonly associated with clinical human cases in several geographical areas. STEC is a significant public health concern. Argentina has the highest incidence of HUS in the world and this study provides the first data about which STEC clones are circulating. Data showed that most of them might pose a serious zoonotic risk and this information is important for developing public health initiatives. However, the actual potential risk will be defined by the virulence profiles, which may differ among isolates belonging to the same ST.

## Introduction

Shiga toxin-producing *Escherichia coli* (STEC) strains are a diverse group of food-borne pathogens, which are responsible for disease in humans such as diarrhea, hemorrhagic colitis (HC) and hemolytic uremic syndrome (HUS). Argentina has the highest incidence of HUS in the world, approximately 500 HUS cases are reported annually and the incidence ranges between 7.8 and 17 cases per 100,000 children less than 5 years of age (Rivas et al., 2010). Ruminants, especially cattle, are the main reservoirs of STEC. Transmission to humans occurs through the ingestion of contaminated food or water, through direct contact with animals or their environment, or via person-to-person transmission (Karmali et al., 2010). *E. coli* O157:H7 is the dominant STEC serotype in the United States, Argentina, Great Britain, and Japan (Abu-Ali et al., 2009). However, multiple reports have shown that non-O157 STEC serotypes, such as serogroups O26, O91, O103, O111, O113, O118, O121, and O145, frequently cause sporadic cases of human illness, and have been implicated in numerous outbreaks (Karmali et al., 2003; Abu-Ali et al., 2009; Bettelheim and Goldwater, 2014). In Argentina, non-O157 serotypes O2:H11, O15:H27, O25:NM, O26:H11, O58:H40; O103:H2, O103:H25, O113:H21, O121:H19, O145:H25, O145:NM, O171:H2, O174:H21, and ONT:NM have been associated with HUS and/or bloody diarrhea in children (Rivas et al., 2006).

The use of nucleotide sequence variation at multiple housekeeping genes has become increasingly popular for strain characterization, as it has advantages for inferring levels of relatedness between strains and the reconstruction of evolutionary events. The introduction of multilocus sequence typing (MLST) has had a marked impact on both epidemiological surveillance and microbial population biology and has revolutionized clonal structure analyses of bacterial populations (Feil et al., 2004; Maiden, 2006). In *E. coli*, the identification of the clones/clonal complexes/phylogroups is crucial, as a strain's ecological niche, lifestyle, and propensity to cause disease varies with its phylogenetic origin (Clermont et al., 2015, and authors cited in it).

At present, we do not know what STEC clones, especially of non-O157:H7, are circulating in Argentina. To fill this knowledge gap we assessed the genetic diversity of STEC strains isolated in Argentina from various sources, mostly cattle and food, using MLST. Our objectives were to determine the phylogenetic relationships among strains and to compare them with strains from different geographic origins, especially with those from clinical human cases, in order to evaluate their potential health risk.

## Materials and methods

### Bacterial strains

We selected 59 STEC isolates belonging to 41 serotypes from a collection of the Laboratorio de Inmunoquímica y Biotecnología (CIVETAN, FCV-UNCPBA, Argentina) collected between 1998 and 2003 in Argentina. They were isolated from foods (*n* = 21), cattle (*n* = 37) and human (*n* = 1) and previously analyzed in relation to the presence of genes encoding for Shiga toxin 1 and 2 (*stx1* and *stx2*), intimin (*eae*), enterohaemolysin (*ehxA*), STEC autoagglutinating adhesin (*saa*) and subtilase-cytotoxin (*subA*) (Parma et al., 2000; Padola et al., 2004; Lucchesi et al., 2006; Sanz et al., 2007; Sanso et al., 2015). The sampling strategy was to choose one or two isolates (when it was possible) from each available serotype, and in this last case, preferably of different origin and/or different virulence profile.

### MLST analysis

The isolates were analyzed by MLST using the EcMLST database for pathogenic *E. coli* curated by the STEC Center at Michigan State University. The allelic profiles of isolates were obtained by sequencing internal fragments of seven housekeeping genes, *aspC* (aspartate aminotransferase), *clpX* (caseinolytic protease), *fadD* (acyl-CoA synthetase), *icdA* (isocitrate dehydrogenase), *lysP* (lysine-specific permease), *mdh* (malate dehydrogenase), and *uidA* (beta-D-glucuronidase). The genes were detected by PCR assay according to the provided protocol in EcMLST (http://shigatox.net/ecmlst/cgi-bin/scheme). Amplicon size was verified by electrophoresis on a 1% agarose gel and PCR products were sequenced (Macrogen, Korea). The different alleles of each gene were numbered and allelic profiles, named sequence types (STs), were determined based on the combinations of seven studied loci (Qi et al., 2004).

### Computer analyses of MLST data

According to the grouping performed by EcMLST database, the sequence types based on the 7 locus MLST scheme were assigned to clonal groups (CGs) (Qi et al., 2004).

On the other hand, a population snapshot was generated from the STs identified and STs from closely related STEC strains downloaded from the EcMLST database (accessed October 10, 2015), based on BURST algorithm, using the eBURSTv3 software (Feil et al., 2004), available at http://eburst.mlst.net. The input data used by eBURST are the STs and their allelic profiles. The first step is to divide the input data (e.g., all the isolates within a MLST database) into groups of STs. These clusters of related STs were grouped into clonal complexes (CCs) in accordance with the default group definition (six of seven shared alleles). For each CC, eBURST identified the ST that is most likely to represent the founding genotype or primary founder. The software includes a simple bootstrap procedure which estimates the degree of support for the predicted founder of a clonal complex, as well as for subgroup founders. Bootstrap confidence values were based on 1000 replicates. Those STs that cannot be assigned to any group are called singletons, e.g., STs which differing in two or more alleles from every other ST in the sample. The output data is displayed as a radial diagram, centered on the predicted founding genotype.

## Results

MLST scheme of 7 housekeeping genes was used to identify and compare the STs of 59 STEC strains of different serotypes isolated mostly from cattle and foods in Argentina. We compared the sequence type data to those in the EcMLST database, and a total of 38 different STs were identified among the set of 59 isolates. Of the 38 STs, 17 (45%) were novel STs, determined in 18 of the 41 serotypes studied (Table 1). The remaining serotypes presented STs that have been registered in the database. In the allelic profiles of the 17 new STs identified, nine new alleles were detected in five housekeeping genes (*aspC, clpX, fadD, mdh*, and *uidA*). The new STs and alleles were submitted to the MLST database.

**Table 1:** **Isolates information, allelic profile, sequence types (ST), clonal groups (CG), and clonal complexes (CC) of strains studied**.

**Serotype**	**Isolate**	**Source**	**Virulence profile^a^**	**Allelic profile^b^**	**ST^c^**	**CG^d^**	**CC**
O2:H5	AM 166-1	Adult cattle	*stx2,saa,subA*	21,24,10,2,1,11,5	**1179**	n.a.	Singleton
O5:NM	T 249-5	Calf	*stx1,eae,ehxA*	20,19,13,4,1,12,1	175	42	850
O8:H16	CM 15-2	Ground beef	*stx1,saa*	32,5,2,206,1,8,12	1066	n.a.	Singleton
O8:H16	FO 164	Adult cattle	*stx1,saa*	32,5,2,206,1,8,12	1066	n.a.	Singleton
O8:H19	3M	Ground beef	*stx1,stx2,ehxA*	4,5,2,111,1,5,1	379	63	230
O8:H19	7M	Ground beef	*stx2,ehxA*	7,5,77,4,1,13,23	**1177**	n.a.	230
O15:H21	FB 11	Adult cattle	*stx2*	**178,187**,13,15,1,1,40	**1156**	n.a.	Singleton
O20:H7	AM 216-1	Adult cattle	*stx1,stx2*	7,5,2,2,1,13,1	140	31	230
O20:H19	HT 1-6	Hamburger	*stx1,stx2,ehxA,saa,subA*	7,5,5,4,3,5,23	92	88	230
O20:H19	AP 28-1	Adult cattle	*stx2,ehxA,saa,subA*	**179**,5,5,4,3,5,23	**1157**	n.a.	230
O20:HNT	T 250-4	Calf	*stx1,eae,ehxA*	10,**188**,46,39,1,5,5	**1169**	n.a.	Singleton
O22:H8	36M	Ground beef	*stx2*	5,5,2,2,1,13,23	145	31	230
O22:H8	HW 1-15	Hamburger	*stx1,stx2,ehxA,saa*	5,5,2,2,1,13,23	145	31	230
O25:H19	FB 68	Adult cattle	*stx2,ehxA*	7,5,77,4,1,8,84	**1170**	n.a.	Singleton
O26:H11	T 246-6	Calf	*stx1,eae,ehxA*	7,8,65,4,1,5,**275**	**1158**	n.a.	106
O26:H11	T 8-1	Calf	*stx2,eae,ehxA*	7,8,2,4,122,5,250	943	n.a.	Singleton
O38:H39	T 246-1	Calf	*stx1,eae,ehxA*	11,190,231,208,19,112,263	1102	n.a.	Singleton
O39:H49	AM 169-3	Adult cattle	*stx1,stx2,ehxA,saa,subA*	7,5,2,101,1,5,1	1138	n.a.	230
O39:H49	AM 165-1	Adult cattle	*stx2,ehxA,saa,subA*	7,5,2,101,1,5,1	1138	n.a.	230
O79:H19	27M	Ground beef	*stx2,ehxA,saa,subA*	7,5,5,2,1,8,3	**1171**	n.a.	None
O79:H19	AM 168-3	Adult cattle	*stx2,ehxA,saa,subA*	7,5,5,2,1,8,3	**1171**	n.a.	None
O88:H21	HW 1-7	Hamburger	*stx1,stx2,ehxA,saa*	7,5,13,2,1,5,3	**1172**	n.a.	Singleton
O91:H21	HAB 14	Hamburger	*stx2,ehxA,saa*	5,5,5,2,3,5,5	89	34	230
O91:H21	AP 16-1	Adult cattle	*stx2,ehxA,saa*	5,5,5,2,3,5,5	89	34	230
O103:NM	AM 202-2	Adult cattle	*stx2,eae,ehxA*	4,2,2,2,1,2,2	119	17	119
O103:NM	T 46-6	Calf	*stx1,stx2,eae,ehxA*	4,2,2,2,1,2,2	119	17	119
O103:H2	FB 94	Adult cattle	*stx1,eae,ehxA*	4,2,2,2,1,2,2	119	17	119
O111:NM	T 9-1	Calf	*stx1,eae,ehxA*	7,8,2,4,1,5,8	106	14	106
O112:H2	2M	Ground beef	*stx2*	5,**196**,2,2,1,**203**,3	**1159**	n.a.	Singleton
O113:NM	15M	Ground beef	*stx2*	5,6,71,2,1,8,1	223	30	230
O113:H21	5M	Ground beef	*stx2,ehxA,saa,subA*	5,6,71,2,1,8,1	223	30	230
O113:H21	AP 97-3	Adult cattle	*stx2,ehxA,saa,subA*	5,6,71,2,1,8,1	223	30	230
O116:H21	CM 3-18	Ground beef	*stx2,ehxA,saa*	5,6,71,25,1,8,1	230	30	230
O117:H7	HT 2-2	Hamburger	*stx2*	5,5,2,2,1,5,80	147	31	230
O117:H7	AP 32-1	Adult cattle	*stx2*	5,5,2,2,1,5,80	147	31	230
O118:H16	T 250-2	Calf	*stx1,eae,ehxA*	7,8,2,2,1,5,8	**1173**	n.a.	106
O120:H19	FS 151	Adult cattle	*stx2,ehxA,saa*	7,5,5,2,1,8,3	**1171**	n.a.	None
O141:H7	AM 167-1	Adult cattle	*stx1,stx2,ehxA,saa,subA*	5,5,5,2,1,5,1	144	36	230
O141:H8	AP 31-1	Adult cattle	*stx1,stx2,ehxA,saa*	5,6,71,2,1,5,1	**1174**	n.a.	230
O141:H8	AP 21-1	Adult cattle	*stx2,ehxA,saa,subA*	5,5,5,2,1,5,1	144	36	230
O145:NM	FB 5	Adult cattle	*stx2,eae,ehxA*	11,13,12,14,9,16,40	78	12	78
O145:NM	AMB 166	Adult cattle	*stx2,eae,ehxA*	11,13,12,14,9,16,40	78	12	78
O145:NM	FB 12	Adult cattle	*stx2,eae*	11,13,12,14,9,16,40	78	12	78
O146:NM	FB 32	Adult cattle	*stx2,eae,ehxA*	11,13,12,14,9,16,40	78	12	78
O157:H7	FB 22	Adult cattle	*stx2,eae,ehxA*	1,1,4,3,2,4,4	66	11	66
O157:H7	GAL 26	Human	*stx2,eae,ehxA*	1,1,4,3,2,4,4	66	11	66
O162:H7	EN 1-4	Hamburger	*stx2*	5,**197**,2,2,1,5,80	**1160**	n.a.	230
O165:NM	T 83-1	Calf	*stx2,eae,ehxA*	68,19,**244**,39,1,17,94	**1161**	n.a.	253
O171:HNT	10M	Ground beef	*stx2*	4,6,2,2,1,2,5	**1175**	n.a.	None
O171:H2	CM 20-7	Ground beef	*stx2*	4,6,2,40,1,2,5	130	n.a.	None
O171:H2	AM 217-1	Adult cattle	*stx2*	4,6,2,40,1,2,5	130	n.a.	None
O171:NM	45M	Ground beef	*stx2*	4,6,2,40,1,2,5	130	n.a.	None
O174:H21	CM 25-12	Ground beef	*stx2*	5,5,5,2,3,5,5	89	34	230
O174:H21	AM 170-3	Adult cattle	*stx1,stx2,ehxA,saa*	59,5,64,40,1,5,1	**1178**	n.a.	150
O175:H8	FB 40	Adult cattle	*stx2*	5,5,2,2,1,5,23	146	31	230
O177:NM	FO 127-3	Adult cattle	*stx2,eae,ehxA*	60,19,13,4,1,12,1	850	n.a.	850
O178:H19	FC 104	Adult cattle	*stx2*	7,5,5,2,1,72,1	**1176**	n.a.	Singleton
O178:H19	41M	Ground beef	*stx2,ehxA,saa,subA*	**179**,5,5,4,3,5,23	**1157**	n.a.	230
O185:H7	CM 22-1	Ground beef	*stx2*	5,5,2,2,1,5,80	147	31	230

a *Virulence profile. Genes taken into account: stx1, stx2, eae, ehxA, saa, subA*.

b *Allelic profile based on MLST of 7 housekeeping genes (aspC, clpX, fadD, icdA, lysP, mdh, uidA). Boldface entries represent novel alleles*.

c Boldface entries indicate novel STs. These sequences were submitted to EcMLST with the name which figure in the column “isolate.”

d *n.a., not assigned*.

**Figure 1:**
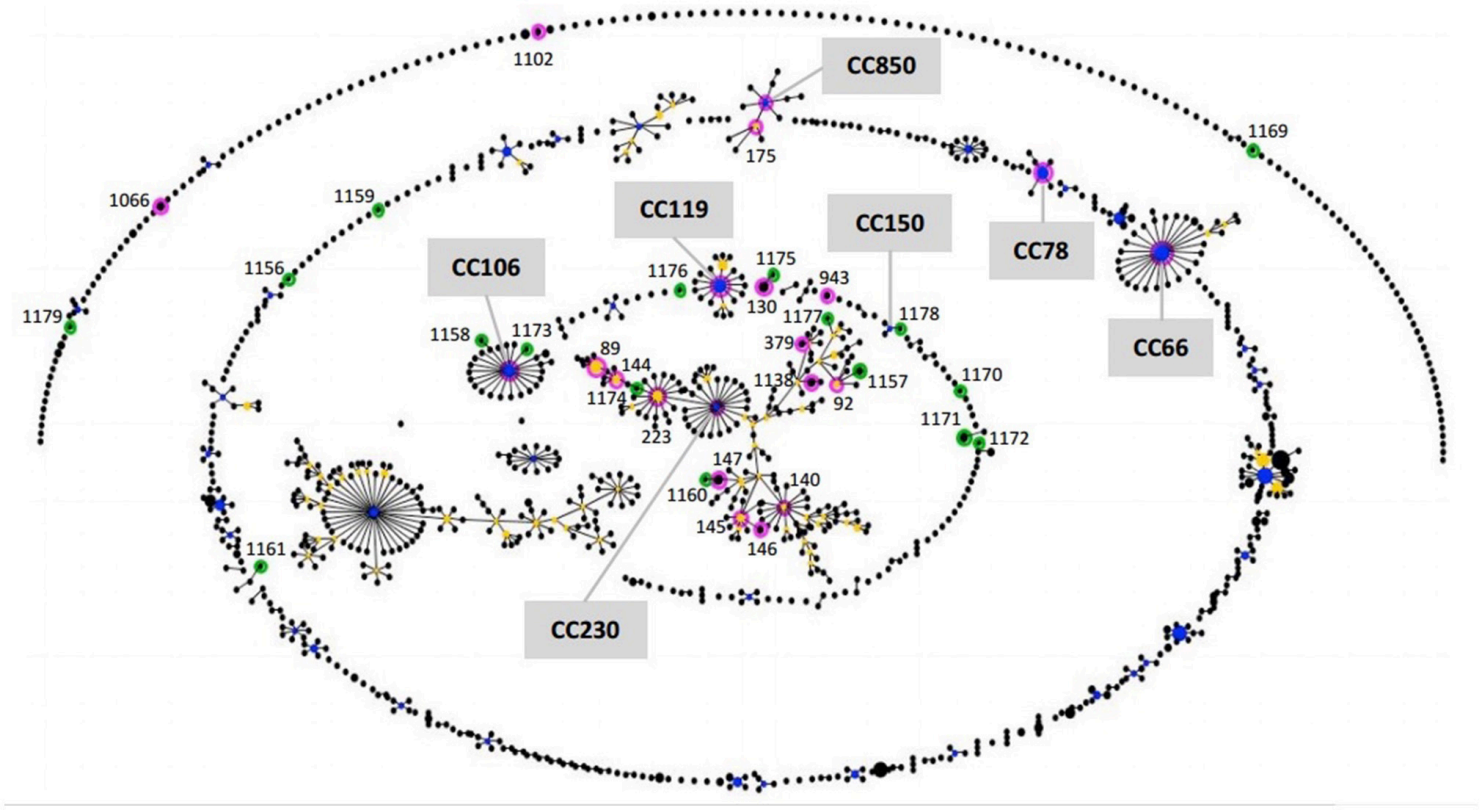
**Population snapshot**. The eBURST diagram shows the clusters of linked STs and unlinked STs in the whole pathogenic *E. coli* population between all ST reported in the EcMLST database (1296 isolates for scheme of 7 genes) and the STs of isolates studied. Each ST is represented as a circle. The frequency of each ST (i.e., the number of isolates of the ST in the input data) is indicated by the area of the circle. The primary founder of the clonal complex is shown in blue, while subgroup founders are shown in yellow. STs in pink and green are those identified in this study. The new STs are in green.

The phylogenetic network displayed by eBURST shown in Figure 1 determined the relatedness between the STs available from the MLST database at the time of the study for scheme of 7 genes (No. isolates = 1296; No. STs = 1112), which includes strains from different countries, the majority from human, and the STs determined in this study.

According to the grouping performed by EcMLST, 15 out of 38 STs identified were clustered into 11 different CGs (Table 1). Of these CGs, six of them grouped strains with similar traits and are defined in the database as CG34 (STEC-1) which included the serotypes O91:H21 and O174:H21, CG30 (STEC-2) including O113:NM, O113:H21 and O116:H21, CG12 (STEC-12) including O145:NM and O146:NM, CG11 (EHEC-1) including O157:H7, CG14 (EHEC-2) including O111:NM, CG17 (EPEC-2) including O103:NM and O103:H2. The other five CGs are conformed by strains with “no common traits” (according to the database). An exception is CG31 which included different O-types, usually associated with H7 or H8 flagellar antigens, such as O20:H7, O22:H8, O117:H7, O175:H8, and O185:H7. The remaining 23 STs, including all new STs found, not grouped in any of the defined CGs.

The eBURST algorithm clustered the input data (STs) into groups (Figure 1), using the most stringent (conservative) group definition (6/7 shared alleles). The isolates in the group defined were considered to belong to a single clonal complex (CC). Only those with bootstrap support higher than 70% (No. re-samplings for bootstrapping = 1000) are taken into consideration in this study. Twenty-four out of 38 STs were assigned to seven different CCs by eBURST. The remaining STs were part of the groups with bootstrap support lower than 35% or grouped in clusters without group founder defined or clustered in distant unrelated STs, as singletons. The clonal complexes (CCs) defined were: CC230, which grouped 15 out of 24 STs determined in this study and comprising 17 serotypes (O8:H19, O20:H7, O20:H19, O22:H8, O39:H49, O91:H21, O113:H21, O113:NM, O116:H21, O117:H7, O141:H7, O141:H8, O162:H7, O174:H21, O175:H8, O178:H19, O185:H7), CC66 (O157:H7), CC106 (O26:H11, O111:NM, O118:H16), CC119 (O103:H2, O103:NM), CC850 (O5:NM, O177:NM), CC78 (O145:NM, O146:NM), and CC150 (O174:H21).

Isolates that belonged to CC230 presented heterogeneity in virulence profiles but none carry *eae* gene. The O157:H7 isolates belonging to CC66 had the same virulence profile (*stx2, eae, ehxA*) but were isolated from cattle and human. All isolates belonging to CC106 had the same virulence profile (*stx1, eae, ehxA*) and were isolated from calves. The three isolates belonging to CC119 had three different virulence profiles (*stx1, eae, ehxA; stx2, eae, ehxA* and *stx1, stx2, eae, ehxA*) and were isolated from adult cattle and calf. The isolates belonging to CC850 had two different virulence profiles (*stx1, eae, ehxA* and *stx2, eae, ehxA*) and were isolated from adult cattle and calf. The isolates belonging to CC78 had two different virulence profiles (*stx2, eae, ehxA* and *stx2, eae*) and were isolated from adult cattle. Only one isolate belonged to CC150 and presented the profile virulence *stx1, stx2, ehxA, saa*.

## Discussion

In order to characterize the phylogenetic relationship of the STEC strains and to assess the potential risk for human infection, we performed a screening of the clones that are circulating in Argentina by analyzing isolates belonging to 41 different serotypes. Although STEC population utilizes similar virulence factors, this group is comprised of phylogenetically different lineages that vary in their ability to cause disease in humans (Abu-Ali et al., 2009).

The different combinations of alleles across seven MLST loci were used to define 38 multilocus genotypes or STs among the 59 strains. In the phylogenetic network, we observed that some of the strains studied were closely related to others of the same or different serotype from previous studies but, however, others showed novel STs.

Whittam (1998) found that STEC strains that are associated with outbreaks and/or hemolytic uraemic syndrome correspond to four clonally related clusters referred to as EHEC-1, EHEC-2, STEC-1, and STEC-2. These four clonal groups differ in their virulence and global distribution (STEC Reference Center, http://shigatox.net/new/about-stec-center/clonal-analysis-of-stec.html). According to EcMLST database, the studied STEC strains were included in 11 clonal groups, among them two EHEC groups and one EPEC group.

EHEC-1 includes serotype O157:H7, its non-motile relatives, and its inferred ancestor O55:H7. The bovine and human O157:H7 strains studied clustered in CG11 like strains from EcMLST database, which were mostly isolated from human in USA and Germany.

EHEC-2 is the most common group of non-O157 Stx-producing strains. It includes strains of several serotypes such as O111:H8 and its non-motile relatives, O26:H11 and O111:H11. These strains clustered in CG14 and the ones included in the database were also isolated from human in USA and Germany.

STEC-1 (CG34) includes many different O types, usually associated with H21 flagellar antigen. These strains typically do not express intimin and do not carry the LEE pathogencity island. O91:H21 and O174:H21 strains studied belonged to this group and the majority of strains submitted at EcMLST were isolated from human and cattle in USA and Canada.

The strains of the group STEC-2 belong to CG30. According to database, this CG is composed of O113:H21 strains, which were isolated in different countries, mainly USA and Canada, and the majority from human. In this study, in addition to O113:H21, O113:NM and O116:H21 strains also belonged to this CG.

On the other hand, and coincidently with results from Isiko et al. (2015), three STEC isolates from cattle belonged to CG17 (denominated EPEC-2), that is not commonly associated with STEC strains, specifically two isolates O103:NM and one O103:H2. Many enteropathogenic *E. coli* strains with H2 antigen isolated from humans in USA and Germany are included in this group.

The CG12, denominated STEC-12 includes O145 strains isolated from human in USA and Germany but, in this study, O146:NM isolate also belonged to this CG.

BURST algorithm uses a model of bacterial evolution in which an ancestral (or founding) genotype increases in frequency in the population to become a predominant clone, and then, begins to diversify, to produce a “clonal complex” (Feil et al., 2004; Spratt et al., 2004). The members of an emerging clone will initially be indistinguishable (same ST) but over time they will diversify to produce a number of variants in which one or two loci have been altered (single or double locus variants [SLVs or DLVs]). The founding ST might be expected to be relatively prevalent in the population, and probably will be geographically more widely disseminated, compared to its descendent variants SLVs and DLVs. Young clonal complexes, such as CC66, will typically have a single strongly predicted founding ST and a number of SLVs and perhaps one or two DLVs. Older clonal complexes will have diversified and are likely to have a less simple structure. In such cases the identification of the founder of the whole clonal complex may be less clear, as both the real founder and the subgroup founders, may have substantial bootstrap support for being the founder (Spratt et al., 2004). CC230 provides an example of a much larger and more diversified clonal complex, with 77% bootstrap support founder and its linked SLVs, some of which have become prevalent and have diversified to form subgroup founders. Indeed, the majority of 24 STs clustered in CCs in this study were grouped within CC230.

Regarding the 17 novel STs identified, 10 were found to be closely related to previously identified STs, of which 6 are included in relevant clonal complexes, STs 1157, 1160, 1174, and 1177 clustered into CC230 (CG30, STEC-2), and STs 1158 and 1173 clustered into CC106 (CG14, EHEC-2). The ST1174 (O141:H8 isolate), particularly, was closely related to ST223 (subgroup founder), differing only in one of the seven MLST loci (SLV). The ST223 includes O113:H21 strains isolated from human (Canada). In the case of STs 1173 (O118:H16 isolate) and 1158 (O26:H11 isolate) have allelic profiles that differ at one and two of the seven loci respectively from ST106 (primary founder), which includes O26:H11 and O111:H8 strains isolated mainly from humans and cattle (USA and Germany).

Among the isolates studied, different STs were found in a same serotype. O8:H19 isolates belonged to two STs, 379 and a novel one, ST1177; O20:H19 isolates belonged to ST92 and a novel ST, 1157; O26:H11 isolates to 943 and a novel ST, 1158; O141:H8 isolates to 144 and a novel ST, 1174; O174:H21 isolates to 89 and a novel ST, 1178; O178:H19 isolates to ST1157 and 1176, both new STs.

We could not determine an association between origin and ST. Seven STs (ST1157, ST1171, ST1066, ST89, ST223, ST147, and ST130) were simultaneously detected among STEC strains isolated from foods and cattle. ST66 (O157:H7 isolates) was assigned to both, a human and a cattle strain. Therefore, and as previously observed in recent studies (Eichhorn et al., 2015; Isiko et al., 2015), there is no clear grouping of certain STs to certain hosts, particularly in populations of non-O157 STEC, but rather the same clone is present in humans as well as cattle or food.

In concordance, too with earlier studies (Abu-Ali et al., 2009; Eichhorn et al., 2015), the resulting MLST revealed that strains of the five most prevalent non-O157 STEC serogroups presented a close relationship; O26, O111, and O118 were clustered together within CC106 and, O91 and O113 were clustered within CC230. Particularly, with respect to the two O26:H11 isolates, they did not cluster together (one of them was a singleton) and none were assigned to ST106, a ST commonly associated with this serotype (Ziebell et al., 2008; Abu-Ali et al., 2009; Contreras et al., 2011). Genetic diversity of O26:H11 has also been demonstrated in previous studies analyzing different genomic regions (Zhang et al., 2000; Bonanno et al., 2015; Krüger et al., 2015).

This is the first study that analyses STEC strains of different serotypes isolated in Argentina by MLST. Therefore, it provides the first data about which clones are circulating in this country. Results highlighted that a number of cattle and food STEC isolates from Argentina belonged to the same STs that are commonly associated with strains from clinical human cases isolated in different geographical regions. We conclude that isolates studied might pose a serious zoonotic risk and, the identifying of high-risk STEC is important for developing public health initiatives. However, the actual potential risk will be defined by the virulence profiles, which may differ among isolates belonging to the same ST.

## Author contributions

This work was carried out in collaboration between all authors. Author JSC performed the laboratory work, analyzed the data and wrote the manuscript; AMS and AVB participated in the planning of the study, drafting and critical review of the manuscript and supervised the work; JG collaborated with the laboratory work. All authors read and approved the final manuscript.

### Conflict of interest statement

The authors declare that the research was conducted in the absence of any commercial or financial relationships that could be construed as a potential conflict of interest.
